# Bibliometric and scientometric analysis of PSMA-targeted radiotheranostics: knowledge mapping and global standing

**DOI:** 10.3389/fonc.2024.1397790

**Published:** 2024-07-01

**Authors:** Mohamed Sallam, Mahan Mohammadi, Frank Sainsbury, Nam-Trung Nguyen, Nobuo Kimizuka, Serge Muyldermans, Martina Benešová-Schäfer

**Affiliations:** ^1^ Queensland Micro- and Nanotechnology Centre (QMNC), Griffith University, Nathan, QLD, Australia; ^2^ School of Environment and Science (ESC), Griffith University, Nathan, QLD, Australia; ^3^ Griffith Institute for Drug Discovery (GRIDD), Griffith University, Nathan, QLD, Australia; ^4^ Centre for Environment and Population Health (CEPH), School of Medicine and Dentistry, Griffith University, Gold Coast, QLD, Australia; ^5^ Department of Applied Chemistry, Graduate School of Engineering, Kyushu University, Fukuoka, Japan; ^6^ Center for Molecular Systems (CMS), Kyushu University, Fukuoka, Japan; ^7^ Research Center for Negative Emissions Technologies (K-NETs), Kyushu University, Fukuoka, Japan; ^8^ Laboratory of Cellular and Molecular Immunology (CMIM), Vrije Universiteit Brussel, Brussels, Belgium; ^9^ Research Group Molecular Biology of Systemic Radiotherapy, German Cancer Research Center (DKFZ), Heidelberg, Germany

**Keywords:** bibliometric and scientometrics, prostate cancer, prostate-specific membrane antigen (PSMA), PSMA-targeted radiotheranostics, PSMA pioneers, leading PSMA

## Abstract

**Purpose:**

Bibliometric and scientometric analyses provide a structured approach to large amounts of data, enabling the prediction of research theme trends over time, the detection of shifts in the boundaries of disciplines, and the identification of the most productive countries, institutions and scholars. In the context of prostate-specific membrane antigen (PSMA)-targeted radiotheranostics, no bibliometric or scientometric analysis has been published thus far. Therefore, this study was conducted to identify key contributors to the literature, assess the global scientific production of related research, and possibly predict future development patterns.

**Methods:**

Scientometrics and bibliometrics were utilized to analyze the current body of knowledge while tracking its evolution to support scientific decision-making comprehensively and systematically. Science mapping techniques were employed to visualize research activities. Two different tools, Tableau and VOSviewer, were utilized, with VOSviewer being deemed the most suitable for the research objectives. The Web of Science (WoS) was used as the principal database for the searches.

**Results:**

Through the search process over a period of 30 years (January 1993–January 2023), 694 original studies in the English language were subjected to comprehensive analysis. By employing bibliometric and scientometric methods, multiple networks were created that mapped various concepts, such as publication trends, leading countries, cocitations, coauthorship among researchers and scientists, as well as coauthorship among organizations and funding agencies. This study revealed the evolutionary patterns, trends, outliers, and key players in the PSMA field, which enabled a more nuanced understanding of the research landscape.

**Conclusion:**

This research contributes to the enrichment of knowledge on PSMA-targeted radiotheranostics through detailed global bibliometric and scientometric analyses. It stresses the necessity for the development of communication platforms, the establishment of supportive infrastructures, and the implementation of proactive solutions to address emerging challenges. This study offers a significant resource for delineating effective strategies and identifying prominent funding bodies essential for continuous advancements in the field of PSMA-based diagnosis and therapy for prostate cancer. It is vital to sustain this momentum to ensure further progress in this pioneering area.

## Graphical Abstract

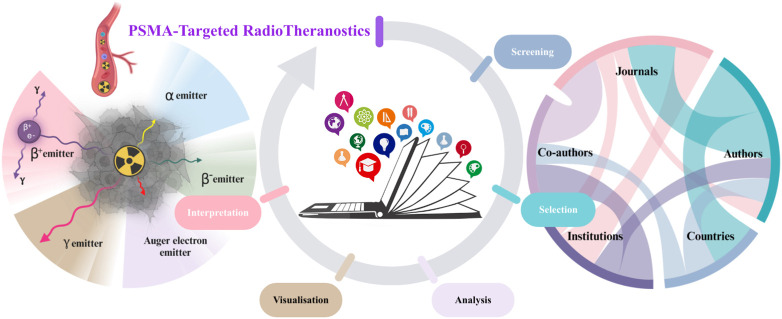

## Introduction

Prostate cancer (PCa) is the most common cancer among men, excluding skin cancer. In 2023, approximately 288,300 men in the United States are expected to be diagnosed with PCa. Globally, about 1.4 million new cases were reported in 2020, making it the fourth most commonly diagnosed cancer worldwide. Incidence rates dropped significantly from 2007 to 2014 due to changes in screening guidelines that reduced prostate-specific antigen (PSA) testing. However, since 2014, overall incidence rates have increased by about 3% annually, with advanced-stage PCa rates rising by 5% each year​ (1). By 2040, the number of annual prostate cancer cases is expected to double, and deaths are projected to increase by 85% to nearly 700,000 annually. This rise is driven by an aging population and lifestyle-related risk factors such as obesity and diet. Significant disparities in incidence and mortality rates exist, with the highest mortality rates seen in countries with limited healthcare access​ (2)​. The PCa death rate has declined by half from 1993 to 2013, thanks to improvements in screening and treatment. From 2016 to 2020, the decline slowed to just over 0.5% per year, likely due to the rise in advanced-stage diagnoses. Currently, there are more than 3.1 million PCa survivors in the United States. Approximately 83% of PCa is diagnosed when the disease is confined to the prostate and nearby organs (70% local and 13% regional). The 5-year relative survival rate for local or regional PCa is nearly 100%, whereas it drops to 32% for cancer that has metastasized​ (1, 2).

Efforts to combat PCa focus on early detection, advanced treatment options, and addressing disparities in healthcare access. While screening guidelines and their effectiveness continue to be debated, lifestyle modifications such as weight management and dietary changes are crucial in reducing risk. These initiatives are essential for lowering incidence and mortality rates globally, highlighting the importance of continued research and public health strategies​ (2). Radiotheranostics, a pivotal aspect of precision nuclear medicine, utilizes radiopharmaceuticals for diagnosis and therapy (3, 4). These compounds consist of radionuclides, functional elements, and biomarker-affine pharmacophores that specifically target cancer cells. Their efficacy hinges on precise radiolabeling and a comprehensive evaluation of their stability, sensitivity, specificity, pharmacokinetics, and tissue distribution (5, 6). Molecular imaging, which leverages tumor biomarkers, surpasses conventional imaging methods such as computed tomography (CT) and magnetic resonance imaging (MRI) in accurately localizing, staging, and restaging cancer (7, 8). A crucial step in this process is selecting patients for targeted radionuclide therapy (TRNT) based on molecular imaging, which also facilitates post-therapeutic monitoring and personalized dosimetry (9, 10).

Bibliometric studies have been conducted to analyze various aspects of PCa research. For instance, a study by Zhong et al. analyzed the global scientific production of PCa immunotherapy (11). This bibliometric analysis evaluated the contributions and cooccurrence relationships of countries/regions, institutions, journals, references, authors, and keywords to identify research hotspots and potential future trends. Other studies conducted a bibliometric analysis of highly cited papers in the subject category of radiology, nuclear medicine and medical imaging, which also included PCa research (12–14). This study aimed to highlight the trends and hot topics in the prostate-specific membrane antigen (PSMA) field, providing awareness and research directions for medical researchers and healthcare practitioners.

### Research insights

This study ventures beyond basic statistics to unearth complex patterns in PCa research, focusing on PSMA-targeted radiotheranostics. It investigates the multifaceted dynamics of this field, uncovering critical outliers, key drivers, and the role of funding in shaping global research trends. By providing a holistic overview, this study identified gaps in the literature, aiding researchers in comprehensive analysis. It also offers a succinct numerical and visual depiction of the global standing of PSMA-targeted radiotheranostics in PCa research.

### Research objectives

An analysis of the Web of Science (WoS) Core database from the past two decades revealed critical topics, influential articles, and prolific authors. By examining the interplay between PSMA and radiotheranostics, this study highlights its pivotal role in PCa and forecasts the future of nuclear medicine research. Although the theme explored in this manuscript does not immediately pertain to clinical scenarios, it resonates with scientific and economic importance, casting a spotlight on the captivating realm of nuclear medicine. This becomes particularly pronounced in ongoing investigations into PSMA-targeted radiotheranostics, weaving a narrative of significance for those navigating the complexities of PCa research.

## Methods

### Data retrieval sources and strategy

The WoS Core Collection, by Thomson Reuters, managed by Clarivate Analytics, serves as the primary database in this study. It is a comprehensive and authoritative source for scholarly publications, widely utilized in scientometric research. The WoS Core Collection includes a broad spectrum of scientific discoveries from diverse disciplines, encompassing articles, meeting abstracts, books, and projects. From 2001 to 2020, there was a significant growth disparity in the Web of Science Core Collection, the SCIE database expanded its paper count by 2.45 times, with citable items increasing 2.64 times, while SSCI tripled its papers and saw 4.45 times rise in citable items. Conversely, A&HCI showed stagnant growth, with only a 71% rise in citable items. Journal growth was robust for SCIE and SSCI, increasing by 48% and 128% respectively, while A&HCI fluctuated, especially post-2010. Gold OA journals surged in SCIE to 20% by 2020, with SSCI and A&HCI lagging at 9% and 7%. The inclusion of individually selected journals notably influenced SSCI and A&HCI, with a policy shift in 2018 leading to their absence by 2020. These disparities underscore the uneven development across disciplines, with A&HCI facing stagnation amidst a backdrop of broader growth in SCIE and SSCI (15). Holding more than 90 million records and a billion references is a crucial resource for obtaining publication and citation information. The credibility of the WoS as a data source for scientometric literature analysis has been validated by numerous studies (16).

VOSviewer has gained prominence among researchers for revealing bibliographic complexities such as coauthorship networks, citations, and keyword maps. Its user-friendly interface and robust functionalities enable swift pattern detection in large datasets, contributing to its widespread acceptance (17). This software significantly impacts bibliometrics and scientometrics, offering crucial insights into research dynamics, collaboration, and trends.

Scientometrics and bibliometrics form the “science of science,” analyzing scientific literature (18). This study merges these approaches to grasp library studies comprehensively and aid informed decision-making. To visualize research activities, science mapping via bibliometrics and scientometrics was used. After careful consideration, VOSviewer, Tableau, and Excel were identified as the most suitable tools for this study due to their strengths in bibliometric analysis, data visualization, and data management, respectively. The literature search was conducted using the following keywords: “Prostate Cancer,” “PSMA,” “Prostate-Specific Membrane Antigen,” “Radionuclide,” and “Nuclear Medicine” in both the title and abstract of the literature. These keywords were chosen to ensure a comprehensive capture of relevant studies in the field of PSMA-targeted radiotheranostics. The search query was constructed as follows:

(TI=("Prostate cancer" OR PSMA OR "Prostate Specific Membrane Antigen" OR "Prostate-Specific Membrane Antigen")) AND TI=(radionuclide OR "Nuclear Medicine") OR (AB=("Prostate cancer" OR PSMA OR "Prostate Specific Membrane Antigen" OR "Prostate-Specific Membrane Antigen")) AND AB=(radionuclide OR "Nuclear Medicine") and Book Chapters or Biographical-Item or Meeting Abstract (Exclude – Document Types) and Physics Condensed Matter (Exclude – Web of Science Categories) and English (Languages).

According to the conclusion of two authoritative literatures studies, the used sub-datasets and coverage years of Web of Science Core Collection should be disclosed to readers (19, 20), as follows:

Web of Science Core Collection (1900-present):

▪ Search the world’s leading scholarly journals, books, and proceedings in the sciences, social sciences, and arts and humanities and navigate the full citation network.
▪ All cited references for all publications are fully indexed and searchable.
▪ Search across all authors and all author affiliations.
▪ Track citation activity with Citation Alerts.
▪ See citation activity and trends graphically with Citation Report.
▪ Use Analyze Results to identify trends and publication patterns.

The list of database editions accessible through the Web of Science subscriptions at Griffith University in 2023 is as follows:

▪ Science Citation Index Expanded (SCI-EXPANDED)—1900-present.
▪ Social Sciences Citation Index (SSCI) –1900-Present.
▪ Art & Humanities Citation Index (AHCI)—1975-present.
▪ Conference Proceeding Citation Index – Science (CPCI-S)—1900-present.
▪ Conference Proceeding Citation Index – Social Sciences & Humanities (CPCI-SSH)—1900-present.
▪ Book Citation Index – Science (BKCI-s)—2005-present.
▪ Book Citation Index – Social Sciences & Humanities (BKCI-SSH) –2005-present.
▪ Emerging Sources Citation Index (ESCI)—2019-present.
▪ Current Chemical Reactions (CCR-EXPANDED)—1985-present.
▪ Index Chemicus (IC)—1993-present.

**Figure 1:**
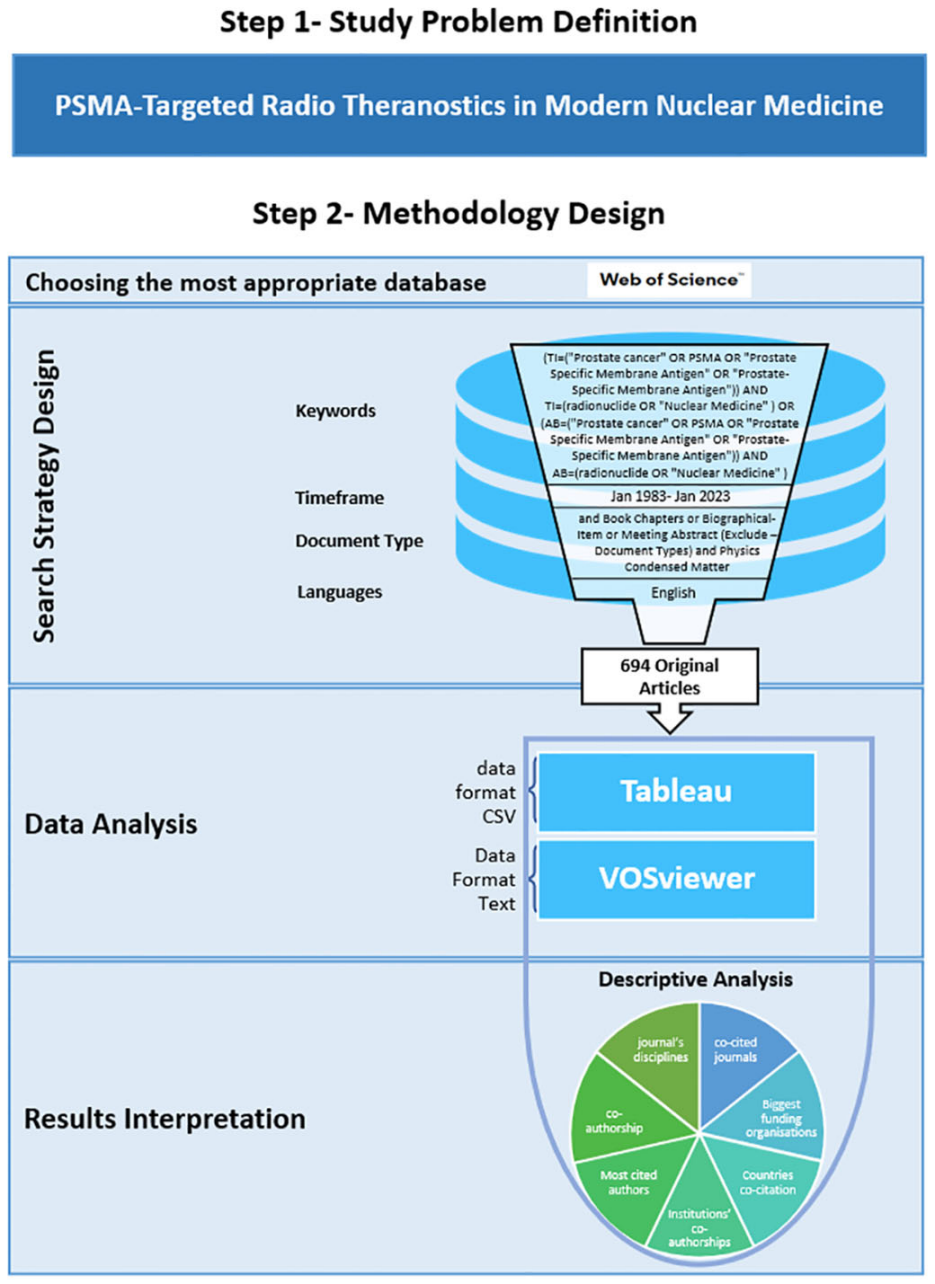
Methodology chart illustrating the key steps of the study, starting with problem definition, followed by search strategy design, software utilized for data analysis, and conclusion with interpretation of the results.

Document types such as book chapters, biographical items, and meeting abstracts were excluded to focus on peer-reviewed articles. Additionally, publications categorized under “Physics Condensed Matter” in the WoS were excluded to eliminate irrelevant entries. The search was restricted to publications in English to maintain consistency and comprehensibility. The data cleaning process involved standardizing terms to avoid duplication, synonyms, and overlaps within the clusters. This was crucial for maintaining the accuracy and coherence of the analysis. Irrelevant publications were filtered out by applying exclusion criteria, ensuring that only pertinent studies were included in the final dataset (**Figure 1**).

### Nature of analysis

Over a period of 30 years (January 1993–January 2023), a search yielded 694 original studies in English, excluding publications in other languages. The application of scientometrics in information science sought to standardize methodologies for determining productivity patterns or, in other words, social activity. The bibliometric section focused on growth in the literature and finding patterns in the database concepts such as publication trends, countries, cocitations, coauthorship, organizational connections, funding agencies, journals, and influential contributors in the field. The generated maps helped identify evolution patterns, gaps, and outliers through both performance and science mapping.

### Definitions of VOSviewer labels

**Cluster:** A cluster is a nonoverlapping group of items on a map, where each item belongs to only one cluster. Clusters are identified by unique numbers and do not necessarily include all items on the map, meaning that some items might not belong to any cluster. Typically, if there is only one cluster, it is labelled Cluster 1; if there are two, they are labelled Clusters 1 and 2.

**Link and total link strength:** A link represents a relationship between two items, such as bibliographic coupling between publications, coauthorship among researchers, or co-occurrence of terms. Each link is given a positive value denoting its strength, reflecting shared references, coauthored works, or term frequencies. VOSviewer, a network visualization tool, often displays links with a minimum strength of one, combining these links and items to form the network.

**Publications:** The number of documents published by a source, an author, an organization, or a country.

**Citations:** The citation attribute is significant when dealing with coauthorship, citation, or bibliographic coupling links. It signifies the number of citations a document has received, or the total citations received by all documents from a particular source, author, organization, or country. In the context of cocitations links, the citation attribute reflects the number of citations made to a referenced source, author, or specific citation.

**Norm. citations:** The normalized citations. It refers to the normalized number of citations received by a document, or the total normalized number of citations received by all documents published by a specific source, author, organization, or country. This metric provides a standardized measure, accounting for variations in citation practices and output across different entities.

**Avg. pub. year:** The average publication year. It denotes either the average publication year of documents in which a keyword or term occurs, or the average publication year of documents published by a specific source, author, organization, or country. This metric offers insights into the temporal distribution of content related to a given keyword or associated with a particular entity.

**Avg. citations:** The average number of citations. This can be for documents containing a keyword or term or for documents published by a specific source, author, organization, or country, providing an indicator of typical impact or recognition.

**Avg. norm. citations:** The average normalized citations. It provides an average measure of impact, considering normalization, whether for documents with a specific keyword or from a particular source, author, organization, or country.

### Methodology limitations

The methodology depicted in **Figure 1** is meticulously designed; however, it is important to acknowledge certain limitations. Solely utilizing the WoS database and focusing on English-language publications could introduce selection bias, potentially leading to the omission of pertinent global research. Research in scientometrics has highlighted that reliance on a single database can result in incomplete coverage of the literature (21). Additionally, language bias is a recognized issue in bibliometric studies, where non-English publications might be underrepresented, thus skewing the results (22). The reliance of the search strategy on specific search terms might also cause key studies to be missed. The crucial steps of data cleaning and synonym consolidation, which are integral to data integrity, are not explained in the figure. Moreover, the employment of sophisticated analytical tools such as VOSviewer and Tableau may lead to potential interpretive biases. Furthermore, the limitation of using the abstract field lies in the potential omission of relevant studies where key terms are not mentioned in the abstract but are discussed in the full text. This approach may inadvertently exclude significant research contributions. However, including the abstract field helps capture a broader range of articles, ensuring that studies which discuss the topic without highlighting it in the title are not overlooked. Despite these limitations, the authors are confident in the significant impact and valuable contribution this research offers to the PSMA-targeted radiotheranostics community.

## Results and discussion

### Publications on PSMA-targeted radiotheranostics have significantly increased since 2015

**Figure 2:**
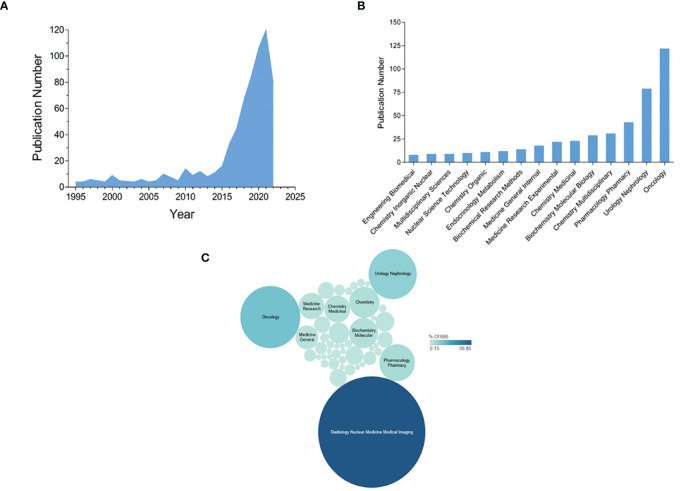
Graphical representations of **(A)** the yearly publication trend in PSMA-targeted radiotheranostics, as recorded in the WoS database, spanning from 1995 to 2022; **(B)** the number of publications per journal; and **(C)** the graphical representation of the number of publications based on the journal’s disciplines.

The analysis of relevant papers revealed a notable surge in PSMA-targeted radiotheranostics research. The data highlighted a consistent upwards trend, signaling heightened interest in this area. This upsurge possibly signifies the growing recognition of PSMA-targeted radiotheranostics as a promising cancer diagnostic and treatment avenue. A screening approach identified 694 pertinent publications in modern nuclear medicine between 1994 and 2022 from the WoS database. **Figure 2A** illustrates a substantial increase in annual publications, particularly since 2015, peaking at 120 publications in 2021. These findings underscore the increasing interest and prominence of PSMA-targeted radiotheranostics as a focal point in cancer research.

The growth of publications on PSMA-targeted radiotheranostics in recent years can be attributed to several key factors. The surge in publications after 2015 stems from multiple factors. Firstly, PSMA is increasingly recognized as a valuable biomarker for PCa due to its high expression in cancer cells and low expression in normal tissues, driving research interest (23). Secondly, advancements in imaging technologies, notably PET/CT and PET/MRI, have significantly improved the precision and accuracy of PCa identification. This has led to interest in developing and utilizing PSMA-targeting radiotracers for diagnosis and radiopharmaceuticals for therapy. Thirdly, the availability of potent radioligands has ignited widespread research across diverse scientific domains, enabling researchers to push the boundaries of medical and biological knowledge.

Additionally, recent clinical trials showing the efficacy and safety of TRNT, particularly in advanced PCa patients, have propelled increased interest and publications in this area (23). Furthermore, the increasing focus on personalized medicine, tailoring treatments based on individual molecular profiles, aligns with the need for more effective and targeted PCa treatments, given its prevalence among men. The confluence of advanced imaging, diverse PSMA-targeted tools, promising trial outcomes, personalized medicine trends, and PCa incidence has significantly elevated publications on PSMA-based radiotheranostics since 2015.

Additionally, greater investment from governmental and private funding bodies has supported extensive research, contributing to the surge in publications (24). According to a recent analysis, the growth in publications can be attributed to the expanding coverage and improved indexing practices of major bibliographic databases like Web of Science (WoS) and Scopus. Scopus, in particular, has a larger coverage of scientific production and a faster indexing process compared to WoS, which allows for the retrieval of more updated data and enhances scientometric analyses with recent publications (15, 25). These databases have become more inclusive and comprehensive over time, capturing a broader range of scientific outputs, and making it easier for researchers to access and publish relevant studies.

### Analysis of journals and cocited journals revealed the most impactful scientific branches

The utilization of journal and cocited journal analysis in mapping PSMA-targeted radiotheranostics knowledge has proven effective in uncovering crucial research themes, influential authors, and institutions within modern nuclear medicine. This analysis serves as a valuable resource for researchers and policymakers, as it aids in informed decision-making, identification of research gaps, and resource allocation. By assessing the global standing and impactful research publications on PSMA-targeted radiotheranostics, researchers can gain insights into the field’s current state and prospective research directions in this promising domain. The highest number of publications was especially common in journals focusing on radiology/nuclear medicine/molecular imaging, oncology, urology/nephrology, and pharmacology/pharmacy (**Figures 2B, C**).

### Germany and the USA are the front-runners in the PSMA research domain

**Figure 3:**
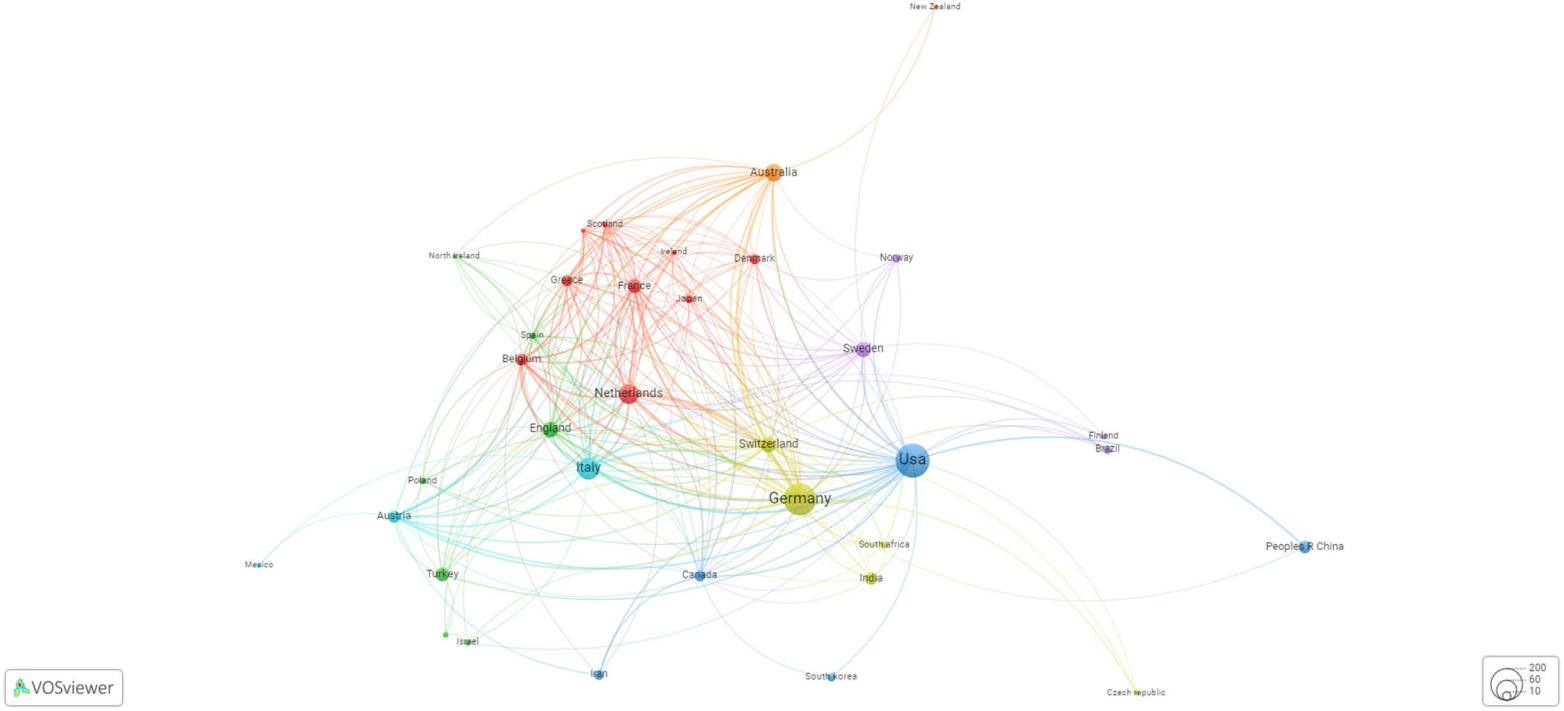
Graphical presentation of collaborative associations between diverse countries based on country cocitations.

In this section, our focus centered on examining the most productive nation’s leading publications in the field. According to **Table 1**, Germany secured the top position in terms of productivity, closely followed by the USA, Italy, Australia, the Netherlands, England, and Switzerland. According to a focused comparison of research impact between Germany and the USA, Germany has become more influential despite the broader network and greater publication volume of the USA. Germany’s total link strength of 216 surpassed that of the USA 158, and its publications received more citations (7,485 vs. 7,118). Moreover, Germany leads in normalized citations (292.60 vs. 214.20) and average citations per publication (44.55 vs. 38.06), indicating a greater impact and contemporary relevance of its research. While the USA has more connections and publications, Germany’s research appears to be quantifiably more influential. By exploring collaborative ties between different countries/regions, **Figure 3** illustrates Germany’s lead in total link strength (TLS), with the USA ranking second. Notably, Italy showcased a strong collaborative bond with Germany. Additionally, it reveals Germany, the USA, Italy, Australia, and the Netherlands as the most prominent collaborative associations based on countries’ cocitations. These nations have established robust partnerships transcending geographic and disciplinary boundaries. Their collaborative research, rooted in shared scientific interests and complementary expertise, reflects the global nature of contemporary scientific endeavors. Through these associations, they leverage collective knowledge to address complex challenges, advance innovation and foster a vibrant, interconnected international research community.

**Table 1:** Top fifty most highly cited authors and nations.

Label	Country	Cluster	Links	Total link strength	Publications	Citations	Norm. citations	Avg. pub. year	Avg. citations	Avg. norm. citations
Haberkorn, Uwe	Germany	2	67	674	16	1787	53.932	2018.1875	111.6875	3.3707
Kopka, Klaus	Germany	2	65	680	15	1623	39.743	2018.4286	108.2	2.6495
Kratochwil, Clemens	Germany	3	65	501	8	1476	32.325	2017.125	184.5	4.0406
Eiber, Matthias	Germany	1	54	349	26	1149	65.3425	2019.6154	44.1923	2.5132
Hofman, Michael S.	Australia	1	60	423	18	1138	48.8591	2019.1111	63.2222	2.7144
Benešová, Martina	Germany	4	58	516	8	1125	27.147	2017.375	140.625	3.3934
Hicks, Rodney J.	Australia	1	58	321	11	1081	35.6607	2018.7273	98.2727	3.2419
Giesel, Frederik L.	Germany	3	65	403	6	1008	20.2306	2017.1667	168	3.3718
Afshar-Oromieh, Ali	Swiss C.	6	65	441	12	957	25.9267	2018.8182	79.75	2.1606
Violet, John	Australia	3	58	254	7	864	26.6776	2018.5714	123.4286	3.8111
Eder, Matthias	Germany	1	59	327	20	803	54.1803	2019.9	40.15	2.709
Herrmann, Ken	USA	2	58	403	8	803	18.9344	2019.000	100.375	2.3668
Iravani, Amir	USA	1	54	196	6	694	21.5539	2018.6667	115.6667	3.5923
Sandhu, Shahneen	Australia	3	55	239	7	679	21.0649	2019.4286	97	3.0093
Bander, Neil H.	USA	3	49	253	7	678	13.9859	2017.5714	96.8571	1.998
Fanti, Stefano	Italy	1	50	224	20	665	41.3226	2019.4	33.25	2.0661
Wester, Hans-Jürgen	Germany	2	62	334	12	656	27.0115	2017.75	54.6667	2.251
Murphy, Declan G.	Australia	1	52	147	5	647	21.1892	2019.8	129.4	4.2378
Morgenstern, Alfred	Germany	6	49	162	6	593	12.345	2016	98.8333	2.0575
Bruchertseifer, Frank	Sweden	6	49	162	5	576	11.937	2017.6	115.2	2.3874
Pomper, Martin G.	USA	3	56	284	16	544	18.4687	2017.6875	34	1.1543
Czernin, Johannes	USA	1	53	171	8	530	33.6105	2019.375	66.25	4.2013
Ceci, Francesco	Italy	1	43	142	8	473	23.9489	2018.75	59.125	2.9936
Rahbar, Kambiz	Germany	1	51	218	8	443	22.2797	2019.125	55.375	2.785
Boerman, Otto C.	Holland	2	38	187	7	439	12.7103	2016	62.7143	1.8158
Umbricht, Christoph	Swiss C.	4	48	328	9	426	17.7662	2018.8889	47.3333	1.974
Schibli, Roger	Swiss C.	4	48	341	9	416	17.5041	2019	46.2222	1.9449
Tagawa, Scott T.	USA	3	47	277	8	373	11.1054	2019.375	46.625	1.3882
Rauscher, Isabel	Germany	1	42	139	9	366	21.3557	2020.3333	40.6667	2.3729
Fendler, Wolfgang P.	Germany	1	53	166	9	356	18.5342	2019.5556	39.5556	2.0594
Maurer, Tobias	Germany	1	35	100	6	353	19.9884	2018.8333	58.8333	3.3314
Heskamp, Sandra	Holland	2	36	186	6	352	11.6624	2018	58.6667	1.9437
Lutje, Susanne	Holland	2	31	109	5	314	9.9349	2015.8	62.8	1.987
Müller, Cristina	Swiss C.	4	36	199	6	310	10.6087	2017.6667	51.6667	1.7681
Ahmadzadehfar, Ho.	Germany	7	46	172	15	308	10.0425	2018.4	20.5333	0.6695
Rijpkema, Mark	Holland	2	28	127	6	305	12.5839	2018.3333	50.8333	2.0973
Gotthardt, Martin	Holland	2	36	98	6	304	11.2573	2018.5	50.6667	1.8762
Rowe, Steven P.	USA	1	42	144	9	291	22.6526	2019.6667	32.3333	2.517
De Jong, Marion	Holland	5	50	201	8	289	9.6996	2017.5	36.125	1.2124
Calais, Jeremie	USA	1	40	104	6	285	13.552	2019.6667	47.5	2.2587
Van Der Meulen, Nic.	Swiss C.	4	37	229	9	277	13.8717	2019.3333	30.7778	1.5413
Mease, Ronnie C.	USA	2	35	77	5	271	4.5133	2014.4	54.2	0.9027
Van Weerden, Wytsk.	Holland	5	50	191	7	271	8.743	2017.2857	38.7143	1.249
Orlova, Anna	Sweden	1	44	99	7	267	10.7426	2017	38.1429	1.5347
Schwaiger, Markus	Germany	5	10	43	10	267	8.5995	2014.4	26.7	0.86
Tolmachev, Vladimir	Sweden	5	10	43	10	267	8.5995	2014.4	26.7	0.86
Babich, John	USA	7	51	146	10	260	9.4437	2017.8	26	0.9444
Essler, Markus	Germany	4	45	151	8	248	6.4542	2018.625	31	0.8068
Baum, Richard P.	Germany	4	34	94	5	218	5.6146	2018	43.6	1.1229
Singh, Aviral	Australia	3	41	103	6	208	9.6656	2018.5	34.6667	1.6109
Gorin, Michael A.	USA	4	48	328	9	426	17.7662	2018.8889	47.3333	1.974

### Top authors with the highest number of citations

**Figure 4:**
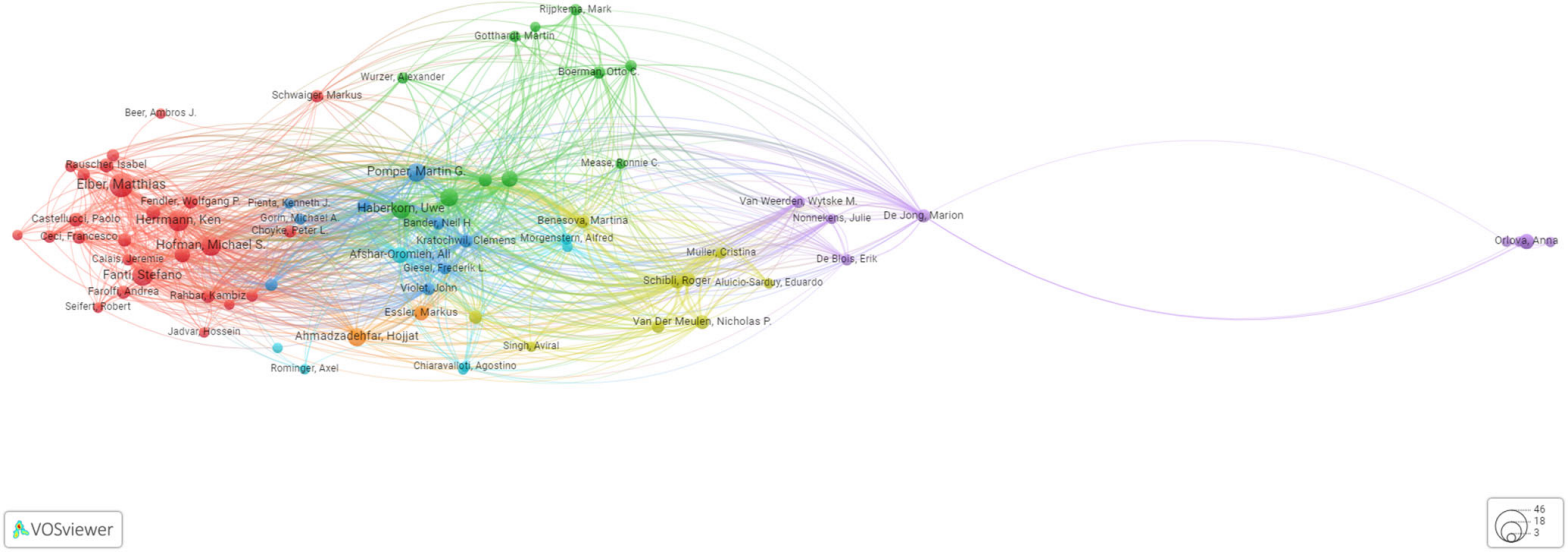
Graphical presentation of the network of authors with more than 40 citations.

The authors’ substantial citation count (**Table 2**, **Figure 4**) underscores their pivotal role in this research field. Cocitations and coauthorship, while distinct, both illuminate intellectual connections among authors. Cocitations reveal research relationships, while coauthorship reflects direct collaboration. Thus, cocitations quantify similarity in research fields, and coauthorship exposes collaborative networks. The data presented in **Figure 4** intriguingly demonstrate that Prof. Anna Orlova of Uppsala University in Sweden is connected to the broader high-citation community through her collaborative work with Prof. Marion De Jong at the Department of Nuclear Medicine, focusing on nuclear biology at Erasmus MC.

**Table 2:** Top 20 most notable coauthors.

Label	Cluster	Links	Total link strength	Citations
Kratochwil, Clemens	3	118	9770	364
Afshar-Oromieh, Ali	1	118	9380	399
Rahbar, Kambiz	3	118	7091	254
Ahmadzadehfar, Hojjat	3	117	5240	179
Hofman, Michael S.	3	117	4543	220
Fendler, Wolfgang P.	1	117	4164	187
Eiber, Matthias	1	117	4022	172
Tagawa, Scott T.	3	117	4004	120
Benešová, Martina	2	117	3593	126
Eder, Matthias	2	118	3463	133
Giesel, Frederik L.	1	117	3457	110
Vallabhajosula, Shankar	2	117	3037	80
Banerjee, Sangeeta R.	2	117	2972	107
Maurer, Tobias	1	117	2874	113
Sathekge, Mike	2	116	2823	88
Baum, Richard P.	3	117	2816	116
Weineisen, Martina	2	118	2658	88
Rauscher, Isabel	1	116	2579	101
Rowe, Steven P.	1	114	2569	98
Bander, Neil H.	2	117	2366	69

### Coauthorship

**Figure 5:**
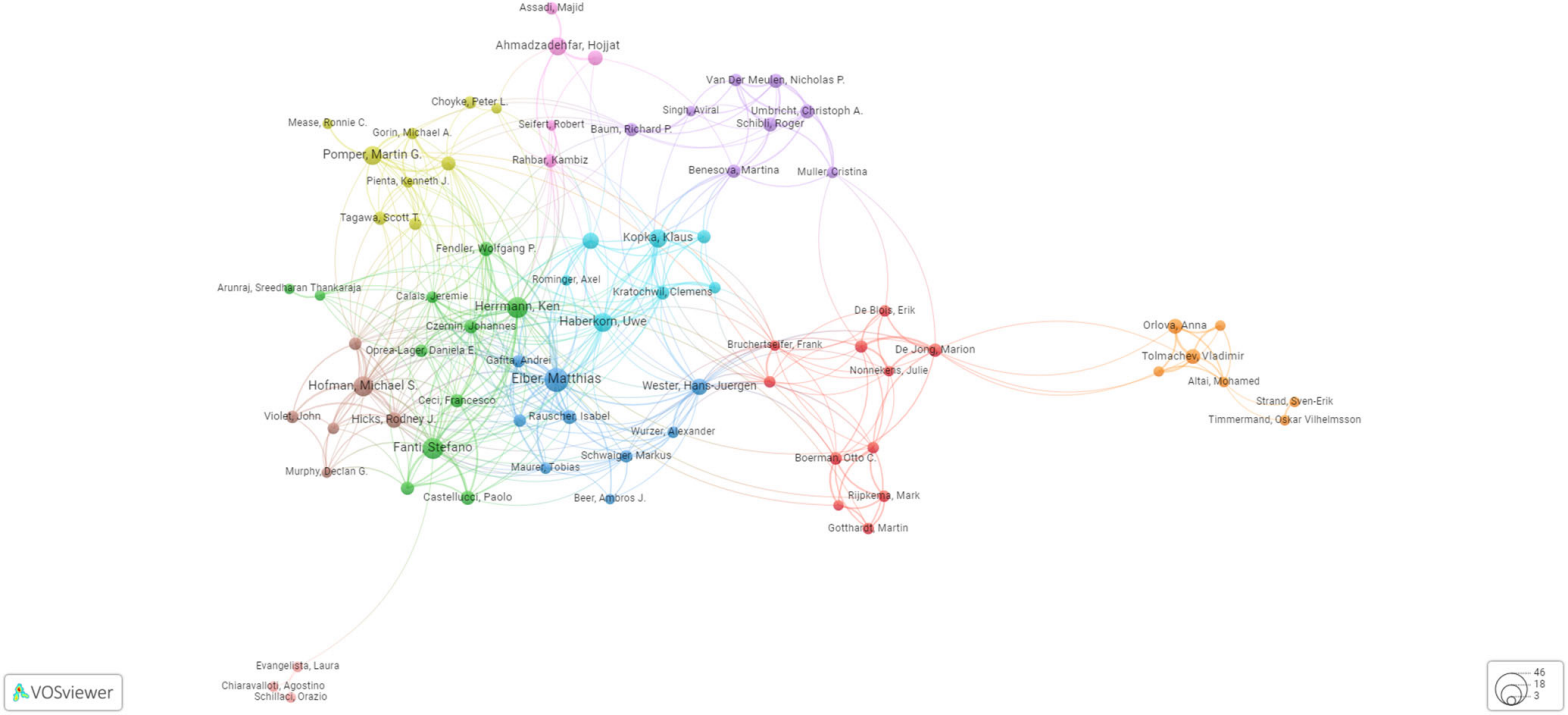
Graphical presentation of the leading coauthors in the domain of PSMA-directed radiotheranostics.

**Figure 5** depicts the top coauthors in the field of PSMA-directed radiotheranostics. The graphic summarizes the most prominent coauthors who have made major contributions to PSMA-targeted radiotheranostics research and publications. Furthermore, **Table 1** lists the most notable coauthors who have made valuable contributions to the field. The tabulated data provide a complete and structured overview of the prominent coauthors who have made important contributions to PCa diagnosis and therapy, enabling rapid and easy comparison of their respective contributions.

### Cocitations

**Figure 6:**
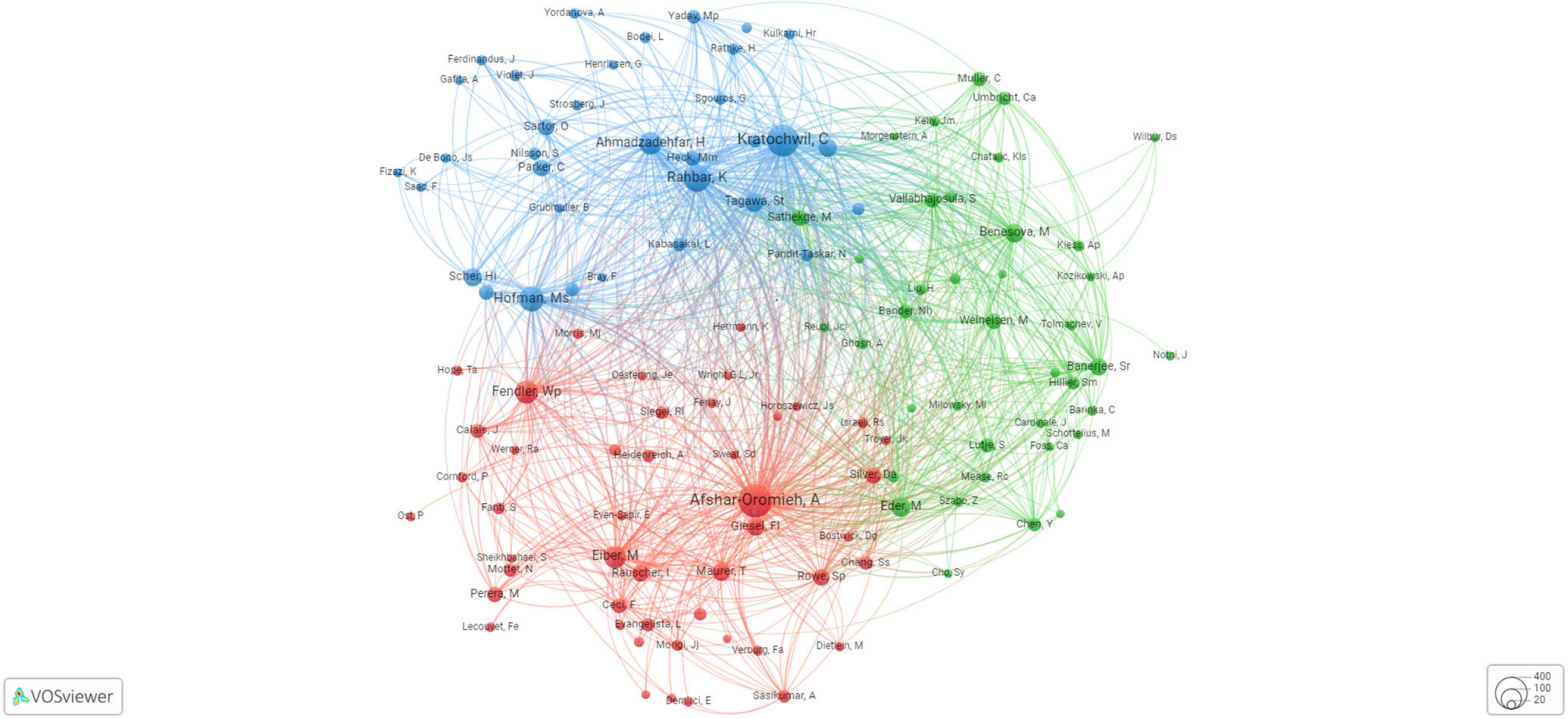
Graphical presentation of the most frequently cited authors on a global scale.

Cocitations are a strategy used to examine the relationship or association between two writers or authors’ research efforts, as described by later writers who have mentioned their work in their own publications. In other words, assessing the frequency with which other scholars cite two different writers’ works on later academic publications is a method of determining the influence and impact of these works. **Figure 6** displays the top 20 and most frequently cited authors globally.

In the context where a substantial portion of noteworthy publications within this domain stem from extensive collaborative accomplishments, it is noteworthy that the impetus behind such endeavors does not consistently emanate from the primary authors. These figures, whose recognition may have been serendipitous rather than stemming from explicit intellectual leadership, adeptly translated their newfound prominence into the construction of a personal profile. Consequently, we deemed it imperative to meticulously examine globally renowned institutions and their influential research groups, particularly those with the greatest impact.

**Figure 7:**
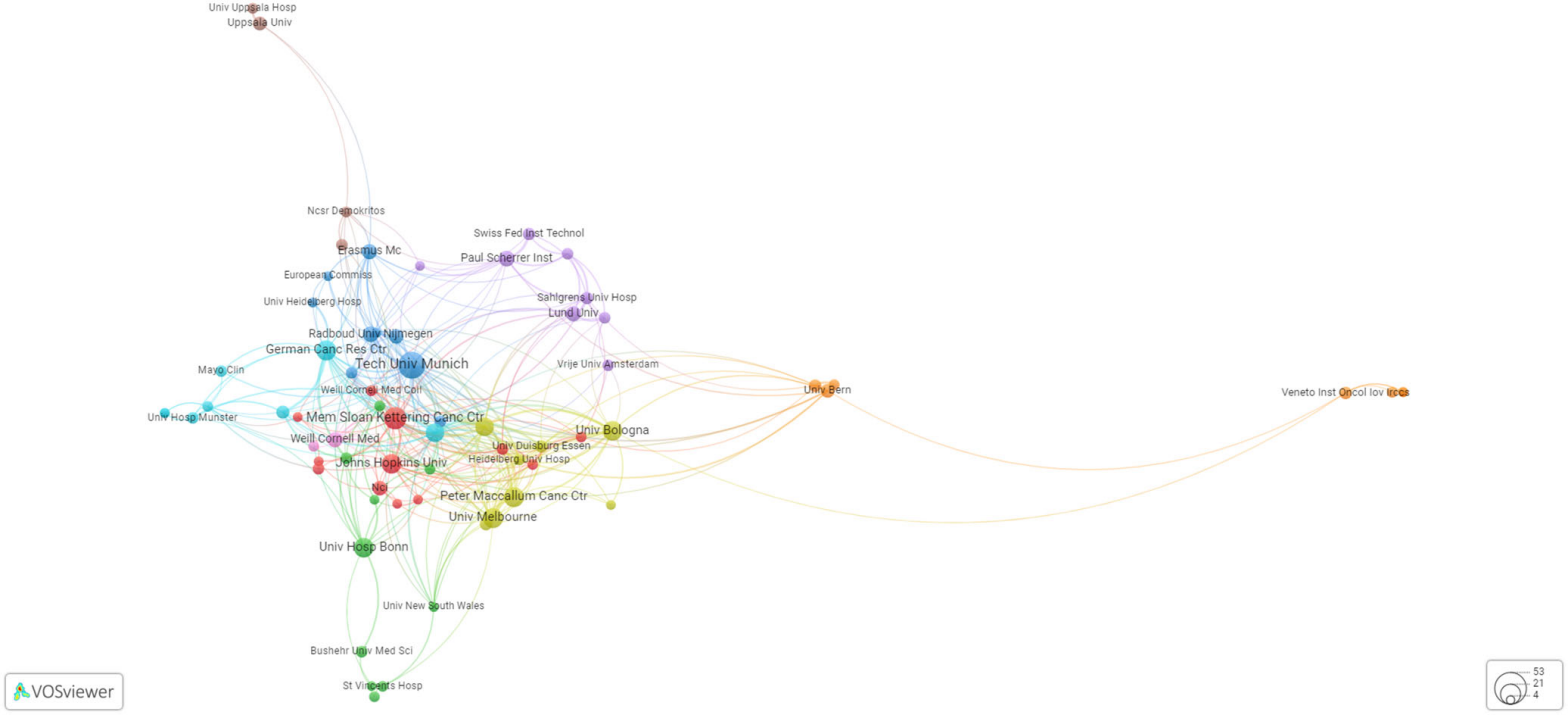
Graphical presentation of the top institutions with the highest coauthorship.

**Table 3** lists the global rankings of research institutions in descending order based on their citation number and authorship. **Figure 7** highlights the top institutions with the most coauthorships. It is expected that most of the world’s best cancer research institutes are in the USA, Germany, Australia, and England, which all have well-established cancer research programs. These countries have invested much time, effort and money in health care, particularly advanced cancer research programs. The Memorial Sloan Kettering Cancer Center (MSK) is famous for its ground-breaking discoveries and innovative therapies for PCa. Similarly, the German Cancer Research Center (DKFZ), in partnership with the University Clinic Heidelberg (UKHD), is globally renowned for its innovative work in advanced PSMA-targeted radiotheranostics applications spanning both basic and translational research, leading to significant discoveries in the field. Moreover, the Technical University Munich (TUM), a well-known university in Germany, has been praised for its excellent research programs for diagnosing and treating PCa. Notably, the Peter MacCallum Cancer Centre (Peter Mac) has played a pivotal role in advancing PSMA-targeted radiotheranostics by conducting groundbreaking research and clinical trials that have expanded our understanding of targeted cancer treatment. Similarly, Weill Cornell University’s contributions in this field have been instrumental, with their innovative studies and collaborations driving forward the development and application of PSMA-targeted radiotheranostics techniques, significantly impacting cancer diagnosis and therapy. Together, these and many other institutions have been key drivers in pushing the boundaries of PSMA-targeted radiotheranostics and enhancing their potential for patient care.

**Table 3:** List of the top 20 institutions with the most coauthorship.

Label	Cluster	Links	Total link strength	Publications	Citations	Norm. citations	Avg. pub. year	Avg. citations	Avg. norm. citations
Memorial Sloan Kettering Cancer Center	2	67	674	16	1787	53.932	2018.1875	111.6875	3.3707
German Cancer Research Center	2	65	680	15	1623	39.743	2018.4286	108.2	2.6495
Technical University of Munich	3	65	501	8	1476	32.325	2017.125	184.5	4.0406
Heidelberg University Hospital	1	54	349	26	1149	65.3425	2019.6154	44.1923	2.5132
Peter MacCallum Cancer Centre	1	60	423	18	1138	48.8591	2019.1111	63.2222	2.7144
University of Melbourne	4	58	516	8	1125	27.147	2017.375	140.625	3.3934
University Hospital Bonn	1	58	321	11	1081	35.6607	2018.7273	98.2727	3.2419
Heidelberg University	3	65	403	6	1008	20.2306	2017.1667	168	3.3718
Johns Hopkins University	6	65	441	12	957	25.9267	2018.8182	79.75	2.1606
The Institute of Cancer Research	3	58	254	7	864	26.6776	2018.5714	123.4286	3.8111
University of California	1	59	327	20	803	54.1803	2019.9	40.15	2.709
Cornell University	2	58	403	8	803	18.9344	2019.000	100.375	2.3668
LM University of Munich	1	54	196	6	694	21.5539	2018.6667	115.6667	3.5923
University of Bologna	3	55	239	7	679	21.0649	2019.4286	97	3.0093
University Hospital Muenster	3	49	253	7	678	13.9859	2017.5714	96.8571	1.998
European Commission	1	50	224	20	665	41.3226	2019.4	33.25	2.0661
Paul Scherrer Institute	2	62	334	12	656	27.0115	2017.75	54.6667	2.251
University of Freiburg	1	52	147	5	647	21.1892	2019.8	129.4	4.2378
Radboud University	6	49	162	6	593	12.345	2016	98.8333	2.0575
Essen University Hospital	6	49	162	5	576	11.937	2017.6	115.2	2.3874

**Figure 8:**
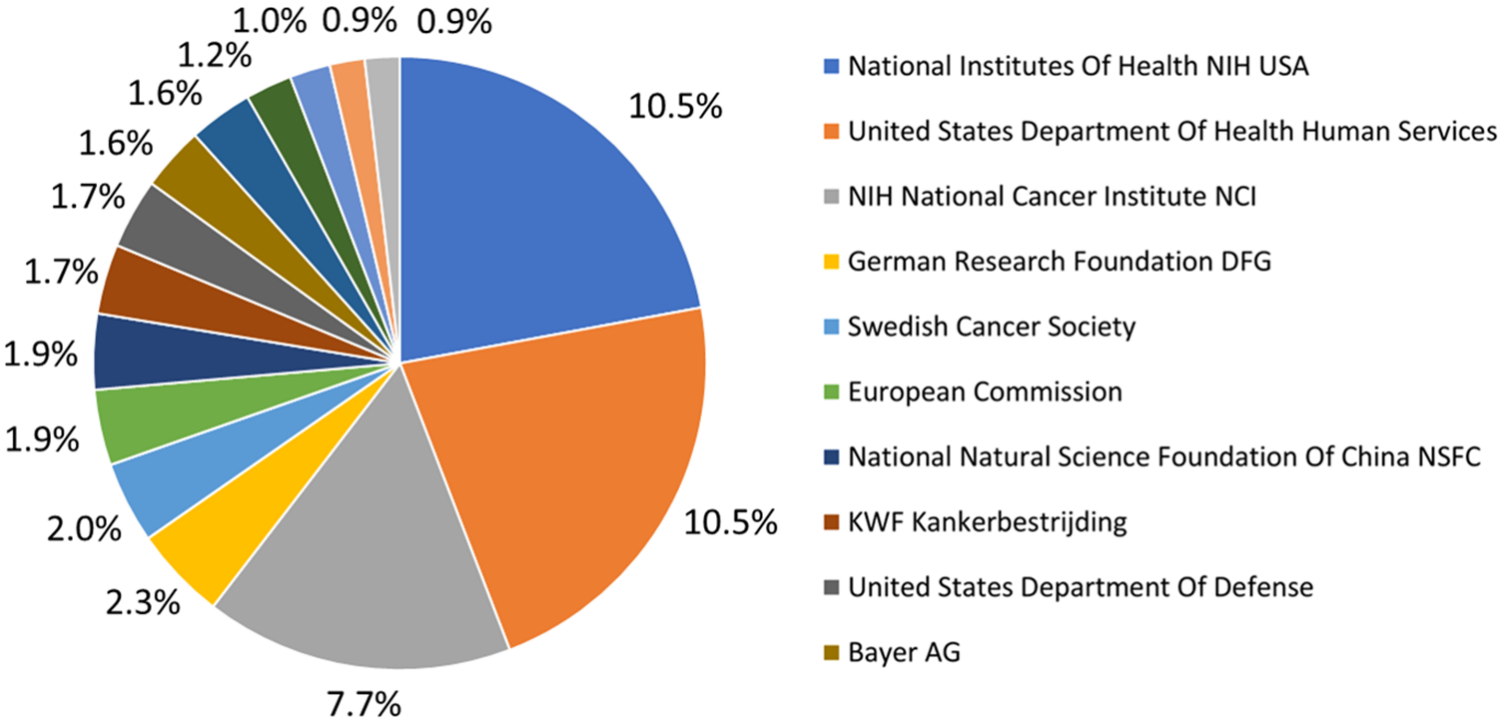
Graphical illustration of the top funding organizations for PSMA-targeted radiotheranostics.

Finally, securing funding for PSMA-targeted radiotheranostics is essential for advancing research and clinical applications. Identifying and accessing appropriate financial resources is a substantial challenge but necessary for the progression of this field. Collaborations fostered by funding bodies can catalyze new insights and drive discoveries across research groups, institutions, and nations. **Figure 8** highlights the principal funding agencies contributing to healthcare advancement. Investment in research and development of novel therapies is crucial for improving patient care and making cutting-edge treatments more affordable and accessible, especially for underserved populations. Specifically, Bayer AG’s investment, shown in **Figure 8**, supports pharmaceutical innovation in PSMA-targeted radiotheranostics, contributing significantly to the evolution of patient treatment and healthcare improvement.

It is important to note the limitations of funding acknowledgment (FA) in the WoS database, researchers have highlighted that WoS funding data can be incomplete or inconsistent, potentially underestimating contributions from various funding bodies (26, 27). Despite these limitations, the use of FA information has increased, and WoS has improved its FA data collection. However, coverage variances persist across indexes by time, language, and document type. Relying solely on FA text is inadequate as many documents report funding information only in specific fields. Articles in Chinese have higher FA presence rates than other non-English publications.

Notably, the Scopus database, increasingly used in academic research, has its own limitations. A case study of 26 English papers published between 2014 and 2019 revealed that WoS collects funding information more accurately than Scopus, which still has errors in funding acknowledgment text and agency fields. Scopus needs to improve its funding acknowledgment text identification and agency extraction strategies (28).

## Conclusion

Although the topic discussed in this manuscript may not possess immediate clinical significance, it holds scientific and economic relevance within the domain of applied nuclear medicine. This research significantly advances our understanding of PSMA-targeted radiotheranostics by employing bibliometric, scientometric, and visual analyses to reveal a substantial increase in global publications, notably from the USA, Germany, Italy, Australia, and England. To fortify this progress, vital efforts include enhancing collaboration among organizations and countries, encouraging academic exchanges, and intensifying cooperation. Creating crucial communication opportunities and supportive platforms for researchers is emphasized. Proactive identification and resolution of potential issues are paramount for ongoing advancements in PCa diagnosis and treatment.

Noteworthy, recent studies, such as the expert review by Fiorentino et al. (2019), have highlighted the significant role of PET/CT in defining radiotherapy (RT) target volumes. The review summarized data for various cancers, including PCa, where PSMA-PET/CT is still under evaluation but shows promising potential. Choline PET/CT has been useful for identifying high-risk volumes in PCa, and FDG-PET/CT has proven crucial for RT planning in other cancers like pancreas, gynecological, and rectum/anal cancers. These molecular and functional imaging approaches represent a major advancement in individualizing RT approaches, improving the precision and effectiveness of treatments (29). Incorporating PSMA-PET/CT into RT planning could enhance the targeting of PCa, potentially leading to better clinical outcomes.

In conclusion, this study has enriched the understanding of PSMA-targeted radiotheranostics for PCa treatment over the past three decades. Serving as a valuable resource for scholars, it highlights credible sources, aids in identifying effective strategies for advancing the field, and spotlights funding organizations. Establishing robust funding and a stable research cycle is pivotal for sustained progress, underscored by this study. Vigilance in sustaining and expanding on the generated momentum is crucial as we strive to push the boundaries of knowledge and improve the lives of those affected by PCa.

## Data availability statement

The original contributions presented in the study are included in the article/supplementary material. Further inquiries can be directed to the corresponding author/s.

## Author contributions

MS: Conceptualization, Data curation, Formal analysis, Investigation, Methodology, Project administration, Resources, Software, Supervision, Validation, Visualization, Writing – original draft, Writing – review & editing. MM: Data curation, Formal analysis, Investigation, Methodology, Software, Visualization, Writing – review & editing. FS: Project administration, Supervision, Writing – review & editing. N-TN: Funding acquisition, Project administration, Resources, Supervision, Writing – review & editing. NK: Supervision, Writing – review & editing. SM: Supervision, Writing – review & editing. MB-S: Conceptualization, Data curation, Project administration, Resources, Supervision, Validation, Writing – review & editing, Formal analysis, Investigation.
